# On the Development of a Computer-Based Tool for Formative Student Assessment: Epistemological, Methodological, and Practical Issues

**DOI:** 10.3389/fpsyg.2018.02245

**Published:** 2018-11-20

**Authors:** Martin J. Tomasik, Stéphanie Berger, Urs Moser

**Affiliations:** ^1^Institute for Educational Evaluation, University of Zurich, Zurich, Switzerland; ^2^Department of Developmental and Educational Psychology, University of Witten/Herdecke, Witten, Germany; ^3^Research Centre for Examinations and Certification, University of Twente, Enschede, Netherlands

**Keywords:** abilities, adaptive testing, competencies, computer-based assessment, education, epistemology, formative assessment

## Abstract

Formative assessments in schools have the potential to improve students’ learning outcomes and self-regulation skills; they make learning visible and provide evidence-based guidelines for setting up and pursuing individual learning goals. With the recent introduction of the computer-based formative assessment systems for the educational contexts, there is much hope that such systems will provide teachers and students with valuable information to guide the learning process without taking much time from teaching and learning to spend on generating, evaluating and interpreting assessments. In this paper, we combine the theoretical and applied perspectives by addressing (a) the epistemological aspects of the formative assessment, with an emphasis on data collection, model building, and interpretation; (b) the methodological challenges of providing feedback in the context of instruction in the classroom; and (c) practical requirements for and related challenges of setting up and delivering the assessment system to a large number of students. In the epistemological section, we develop and explicate the interpretive argument of formative assessment and discuss the challenges of obtaining data with high validity. From the methodological perspective, we argue that computer-based formative assessment systems are generally superior to the traditional methods of providing feedback in the classroom, as they better allow supporting inferences of the interpretive argument. In the section on practical requirements, we first introduce an existing computer-based formative assessment system, as a case in point, for discussing related practical challenges. Topics covered in this section comprise the specifications of assessment content, the calibration and maintenance of the item bank, challenges concerning teachers’ and students’ assessment literacy, as well as ethical and data-protection requirements. We conclude with an outlook on possible future directions for computer-based formative assessment systems and the field in general.

## Introduction

Educational research has experienced a remarkable progress in the past 20 years. This is reflected in the creation of new institutional structures, a massive expansion in funding, and an increase in the public interest and recognition (Köller, 2014). These successful developments can partly be attributed to methodological shifts toward quantitative method. This method has allowed measuring the outputs and outcomes of entire educational systems—a process often referred to as ‘educational monitoring’ (Scheerens et al., 2003). Although educational evaluation results were initially prepared for the use of teachers, principals, and school administrators, it soon became clear that the formative assessment could have a substantial impact on students’ learning and performance (e.g., Hattie and Timperley, 2007). Formative assessments provide feedback on students’ learning progress, encouraging a systematic use of data. The expansion of information technologies has given schools the opportunity to develop an efficient and user-friendly culture of formative assessment for teachers who may not be experts in rigorous test analyses (Brown, 2013), allowing them to focus on teaching. Experts have even argued that an automated formative assessment is the most effective use of digital technologies in the classroom, compared with the other cases of computer-assisted instruction, such as drill-and-practice applications (e.g., Moser, 2016). Technological assessment systems have several advantages for everyday use that make learning visible to students and teachers. Computer-assisted formative assessment helps teachers to focus their attention on instruction and grade data objectively with minimal time and effort expended in data collection and analysis. In addition to assessment for learning and diagnostic testing (see van der Kleij et al., 2015), this data-based decision making in education (see Schildkamp et al., 2013) is considered one of the three most important approaches to the formative assessment. Decisions based on objective data can also increase teaching effectiveness and minimize bias (see Lai and Schildkamp, 2013; Schildkamp and Ehren, 2013).

This paper discusses the core aspects of data-based formative assessment technology. It comprises five parts. In the first part, we provide an overview of the theoretical foundations of the formative assessment, along with some empirical evidence on its benefits for learning. In the second part, we focus on the epistemological aspects of the formative assessment systems and develop an interpretive argument about scoring, generalization, extrapolation, and implication in the formative assessment. In the third part, we examine the methodological challenges of such systems and argue that computer-based technology can provide more effective solutions than the traditional methods. In the fourth part, we introduce a sample case of a computer-based formative assessment system and discuss some fundamental practical requirements related to its development and operation. We conclude with a discussion of possible further developments in computer-based formative assessment and examine some ideas on how it could evolve.

## An Overview of the Formative Assessment Benefits

From a theoretical perspective, formative assessments pursue several purposes. They can ‘provide feedback and correctives at each stage of the teaching-learning process’ (Bloom, 1969, p. 48). They can help us to ‘adapt the teaching to the student needs’ (Black and William, 1998, p. 140). They can also help us to ‘adjust ongoing teaching and learning to improve students’ achievement of intended instructional outcomes’ (McManus, 2008, p. 3). New Zealand’s Ministry of Education (1994) defines formative assessment as ‘a range of formal and informal procedures […] undertaken by teachers in the classroom as an integral part of the normal teaching and learning process in order to modify and enhance learning and understanding’ (p. 48). Given these definitions, most educators and researchers would agree that the formative assessment should not be limited to single tests, but rather needs to be considered an ongoing process (Popham, 2008; Shepard, 2008). This process consists of a cyclical feedback loop in which (a) the students’ current proficiency level is assessed, (b) the assessment-based learning goals are defined, (c) the students’ learning progress is monitored by further assessments, and (d) the learning goals and environments are adjusted based on the assessment outcomes (van der Kleij et al., 2015; see also Brookhart, 2003, p. 7).

The conceptual strength of the formative assessment is to make learning visible (see Havnes et al., 2012). It can also aid in using students’ strengths and weaknesses to frame appropriate learning goals, monitor their progress toward the goals, and to inform the extent of their success or failure in achieving the goals. In essence, the process concerns three fundamental questions: ‘Where am I going?,’ ‘How am I getting there?,’ and ‘Where to go next?’ (Hattie and Timperley, 2007). The answers can be found in the objective data from the assessments. The process can either directly support learning and self-regulation or be used for diagnostics and data-driven decision making (van der Kleij et al., 2015). It also suits the notions of individualization and differentiated instruction (see Levy, 2008). In fact, the formative assessment can be a prerequisite for individualization and differentiation, as it specifies a student’s current standing and her/his extent of progress. The formative assessment is also highly compatible with the current trend toward educational measurements. On the conceptual level, summative and formative assessments share an orientation toward educational outcomes and both can support teaching and learning (Bennett, 2011). On the methodological level, measurement theories that are used include: item-response theory (IRT; see de Ayala, 2009), measurement concepts such as adaptive testing (see Wainer, 2000), and measurement tools such as computer-assisted assessment (see Conole and Warburton, 2005).

There is ample empirical evidence that feedback can substantially benefit learning and self-regulation (e.g., Cawelti and Protheroe, 2001; Campbell and Levin, 2009; Lai et al., 2009; Carlson et al., 2011). Feedback is even considered ‘the most powerful single moderator that enhances achievement’ (Hattie, 1999). The first studies dating back to the 1950s (e.g., Ammons, 1956), and the more recent meta-analyses, suggest remarkable effect sizes. One of the most comprehensive meta-analyses to date was published by Kluger and DeNisi (1996). They collected 607 effect sizes from 131 studies on the effectiveness of feedback interventions on learning and extracted an average *d* = 0.41, which corresponds to a small-to-medium effect size (Cohen, 1992).

In the late 1990s, Hattie (1999) published a synthesis of over 500 meta-analyses involving over 400,000 effect sizes from 180,000 studies on various influences on student achievement. The average effect of schooling was *d* = 0.40 per school year, which can be considered a benchmark against which the effects of feedback can be judged. In sum, 12 previous meta-analyses evaluating 196 studies and almost 7,000 effect sizes were considered. The average effect size was *d* = 0.79, almost twice the average effect of schooling and large (Cohen, 1992). However, there was considerable variability in the effect sizes, depending on the type of feedback provided. For example, the effect sizes of praise (*d* = 0.14), punishment (*d* = 0.20), and reward (*d* = 0.31) were low, whereas receiving feedback related to a specific task (*d* = 0.95) and providing cues on how to solve a problem more effectively (*d* = 1.10) provided the highest effect sizes (see also Hattie and Timperley, 2007).

Empirical evidence concerning effects on self-regulation is less conclusive, although it is widely believed that appropriate feedback should enable the students to monitor the attainments of their learning goals more autonomously (Bernhardt, 2003; Earl and Katz, 2006; Love, 2008; Herman and Winter, 2011). Butler and Winne (1995) suggest that ‘research on feedback and research on self-regulated learning should be tightly coupled’ (p. 245). Overall, studies show positive effects on motivational, metacognitive, and strategy-use aspects of self-regulation with substantial effect sizes (e.g., *d* > 1.00 in Dignath et al., 2008), with the feedback type playing a decisive role (e.g., Nicol and Macfarlane-Dick, 2006).

However, not all studies, reviews, and meta-analyses show positive effects of the formative assessment (or feedback, more specifically) on achievement and self-regulation. Rather, the variability in effect sizes is very large, which points to the possibility of substantial moderation by variables that are still poorly understood. Bennett (2011) argues that the studies usually used in meta-analyses might be ‘too disparate to be summarized meaningfully’ (p. 11). Indeed, 38% of the effects of all studies compiled by Kluger and DeNisi (1996) were *negative*, suggesting higher performance in the control group (see Shute, 2008; Dunn and Mulvenon, 2009; Bennett, 2011).

## Formative Assessment Systems: Epistemological Aspects

**FIGURE 1:**
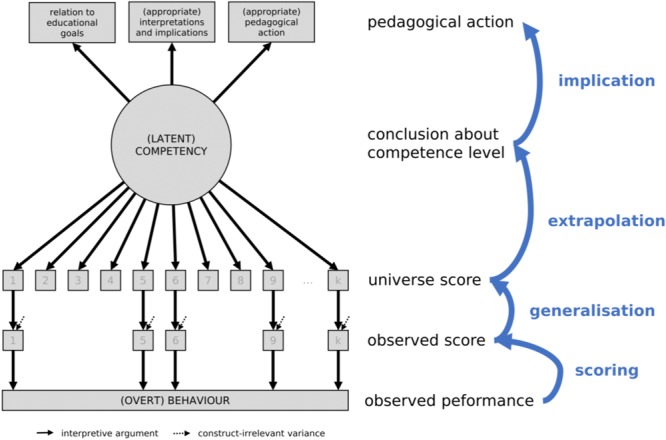
Interpretive argument for formative assessments.

As opposed to more traditional approaches to validity and validation (e.g., Cronbach and Meehl, 1955), the current authoritative approach is that of ‘validity as an argument’ (see Figure 1), in which it is not the validity of a test *per se*, but rather the validity of the meaning of test scores and their implications for action that are evaluated (Kane, 2006, 2013; see also Messick, 1989, 1995). Many alternative concepts of validity exist (e.g., Borsboom et al., 2004), and there is an ongoing substantial debate about the relation between validity and truth (e.g., Borsboom et al., 2004; Kane, 2013; Newton and Baird, 2016; for an overview, see Cizek, 2012; Newton and Shaw, 2014). An in-depth discussion of this debate is beyond the scope of this paper; however, we would like to concentrate on the concept of ‘validity as an argument,’ as a widely accepted notion.

At the core of the concept of validity as an argument is the *interpretive argument*. This can be considered a scientific mini-theory that merits assessment/testing developers’ attention. Interpretive argument should be distinguished from the *validity argument*. This latter argument provides an evaluation of the interpretive argument in terms of clarity, consistency, plausibility, and empirical examination. In other words, the interpretive argument is defeasible by failure in the validity argument, and, as with the other scientific theories, such failure can result in the reformulation, restriction or even rejection of the interpretive argument. In the following section, we will develop an interpretive argument for formative assessment by addressing the issues of scoring, generalization, extrapolation, and implication.

### Scoring Argument

The interpretive argument for the formative assessment comprises four inferences, namely scoring, generalization, extrapolation, and implication (see Table 1). The *scoring inference* is concerned with obtaining valid observed scores from an observed performance. In technical terms, this refers to translating a response, such as a selected multiple choice category or an essay, into a score by means of a scoring key or rating scheme. The scoring rule to do so needs to be free of bias and applied accurately and consistently across all subjects and measurement occasions. This is usually facilitated when standardized tests are used; however, issues may arise when humans are involved in judging performance. In general, the scoring inference for the formative assessment is not much different from those applied to trait interpretations, summative assessments, or placement systems (see Kane, 2006, for more details).

**Table 1:** Interpretive argument for formative assessments.

**Scoring** (from observed performance to observed score)		
S1	Scoring rule is appropriate.	
S2	Scoring rule is applied accurately and consistently.	
S3	Scoring is free of bias.	
S4	Data fit the scaling model employed.	
**Generalization** (from observed score to universe score)		
G1	The sample of observations is representative of the universe of generalization.	
G2	In case of adaptive or tailored testing, parameter invariance holds.	
G3	The sample of observations is large enough to control random error.	
**Extrapolation** (from universe score to conclusion about competence level)		
E1	The universe of generalization is representative of the competency.	
E2	There are no construct-irrelevant sources of variability that would seriously bias the interpretation of the competence level.	
E3	For extrapolations onto higher aggregate levels (e.g., classes), clear participation rules have been followed.	
E4	For extrapolations over time (in terms of learning progress), the learning function must be known.	
**Implication** (from conclusion about competence level to pedagogical action)		
I1	The competence level can be related to an educational goal (‘Where am I going?’).
I2	The implications associated with the competence level are appropriate, and the semantic interpretation of the
I3	competence level is plausible, legitimate, and accurate (‘How am I getting there?’).
I4	Whichever pedagogical action is most appropriate depends on the achieved competence level (‘Where to go next?’).
I5	The decision rules for pedagogical action are appropriate.
	The pedagogical actions taken are effective in improving learning.

*Table partly adapted from Kane (2006).*

### Generalization Inference

The observed scores are based on a sample of observations and a subset of what Kane (2006) labeled the ‘universe of generalization.’ For example, if the sample of observations contains a set of four items, covering bridging to ten in summation, then the universe of generalization would be all the possible items covering this topic (e.g., ‘7 + 5 =,’ ‘7 + 6 =,’ etc.). Hence, the *generalization inference* is concerned with obtaining a valid universal score from the observed score, an issue that is also discussed in more traditional approaches to validity (e.g., Linn et al., 1991). There are three main issues related to generalization. First, the sample of observations needs to be representative of the universe of generalization and, especially in cases of adaptive or tailored testing, parameter invariance must hold (e.g., Rupp and Zumbo, 2006). Ensuring representativeness is best achieved when the universe of generalization is known and a random sample of items is drawn from this universe. However, in reality, the universe of generalization is only known, if at all, for narrowly circumscribed topics (e.g., bridging to ten) and is not well-defined for more complex ones (e.g., writing an argumentative essay). In combination with the context-specific nature of learning and thinking (see Greeno, 1989), this makes the selection of items a challenging endeavor. Second, if a measurement model is employed for scaling, which is almost always the case in computer-based assessments, data need to sufficiently fit the model and its assumptions. This is tested routinely in models based on IRT (e.g., Orlando and Thissen, 2000). However, differential models between relevant subgroups (e.g., boys and girls) are not considered extensively. In some cases, this might represent a threat to test fairness and jeopardize the interpretation of inter-individual and group differences. Biased parameter estimates might also arise when unidimensional models are set up but the measured characteristic is not unidimensional (see Ackerman, 1989). This can be the case when the underlying scales are supposed to cover many or even all the school grades. Finally, the number of observations also needs to be large enough to control for random error. This is particularly difficult to achieve in the formative assessment, in which testing time is usually constrained, and only a limited number of items can be presented at any one time.

### Extrapolation Inference

The next step in the interpretive argument is the *extrapolation inference* from the universe score to a conclusion about the students’ competence levels. For formative assessments, there are three requirements for a valid extrapolation. First, it is necessary that the universe of generalization is representative of the competency or the competency domain to be measured. For example, the universe of all the possible items covering bridging to ten must be representative of the competency to add numbers in the range up to 20. Again, for narrowly described competency domains, this is sometimes self-evident, whereas for more complex domains, this requires more justification. The issue of representativeness in extrapolation has been discussed elsewhere in more detail. For example, Messick (1995) points to the utility of ‘task analysis, curriculum analysis, and especially domain theory’ (p. 745) for defining the structure and content of the competency or its domain. Labeling it as ‘construct domain,’ Messick highlights the importance of covering all parts of the construct domain, which can be achieved through ecological sampling, already suggested by Brunswik (1956). This ‘content coverage’ (Linn et al., 1991) or ‘scope’ (Frederiksen and Collins, 1989) seems to be particularly relevant in the context of the formative assessment, as gaps in coverage might result in students and teachers underemphasizing those parts of the content that were not considered for assessment. Second, it is equally important that what is captured are only the sources of variability relevant to the targeted competency or its domain, which otherwise would seriously jeopardize the interpretation of the competence level. Construct-irrelevant variability tends to contaminate the task by making it either ‘too easy’ or ‘too difficult’ for some students but not for the others. For instance, some items that test the ability of bridging to ten might be color-coded and thus be unduly difficult for color-blind children. Other items might use gender-specific illustrations, thereby eliciting more response from one gender group than from the other. There are many sources of construct-irrelevant variance (see Messick, 1995; Kane, 2006), and they become particularly relevant in the low-stakes testing context of the formative assessment. This is because students tend to reduce test-taking effort in low-stakes assessments, presumably because doing well on the test will bring them limited attainment, intrinsic or utility value for them (Wise and DeMars, 2005). Consistent with the expectancy-value model of achievement motivation (e.g., Wigfield and Eccles, 2000), most research clearly shows that test score validity falls with decreasing test-taking effort, which in turn means that the construct-irrelevant variance and/or error variance more strongly determine the test score. To the best of our knowledge, no extant research has systematically investigated these aspects or has estimated their effects on the validity of formative assessments. We can only speculate that factors such as self-regulation abilities, attention span, conscientiousness at the individual level, classroom climate, availability of computers in the classroom, or teacher support at the system level might be more optimal for some students but not for the others, hence the possibility of construct-irrelevant variance when test-taking effort decreases. Third, teachers or administrative authorities might want to use the formative assessment data to extrapolate a single student’s scores of competence to those of groups of students or the entire student population. This can be problematic in the absence of clear participation rules, causing self-selection bias to affect the estimated competence level. At the very least, information is needed about the (non-)participants in formative assessments, and about how these two groups differ in terms of ability and learning progress. To ensure a valid extrapolation from the universe score to conclusions about the competence level, we require broad representativeness, low construct-irrelevant variability, and participation transparency.

### Implication Inference

The final step in the interpretive argument is the *implication inference* from the competence level to pedagogical (or administrative) action. Assessment experts consider this step the most important yet the least controllable. It is essential to note that some definitions of the formative assessment *always* encompass a strong functional element. For example, New Zealand’s Ministry of Education (1994) defines the formative assessment as ‘a range of formal and informal procedures […] undertaken by teachers in the classroom as an integral part of the normal teaching and learning process *in order to modify and enhance learning and understanding*’ (p. 48, emphases added). Brown and Cowie (2001) define it as ‘the process used by teachers and students to recognize and respond to student learning *in order to enhance that learning* during learning’ (p. 510, emphases added); they further argue that ‘assessment can be considered formative only if it results in *action by the teacher and students* to enhance student learning’ (p. 539, emphases added). Finally, for Black and William (1998), ‘assessment becomes ‘formative’ when the evidence is *actually used to adapt the teaching*’ (p. 140, emphases added). Hence, if the purpose of the formative assessment is to enhance learning, then validity is about whether this purpose is achieved or not (see Stobart, 2012). This notion of consequential validity was first proposed by Messick (1989, 1995) and further developed by Kane (2006, 2013), both of whom focused strongly on the uses (and misuses) of test scores in theorizing about validity and validation.

The implication inference in formative assessments comprises five aspects (see Table 1). The first three facets refer to the central functions of the formative assessment, as identified by Hattie and Timperley (2007), whereas the latter two address issues of effectiveness, and whether they instigate the appropriate pedagogical action. Because the purpose of the formative assessment is to ‘reduce discrepancies between current understandings/performance and a desired goal’ (Hattie and Timperley, 2007, p. 87), an effective formative assessment needs to meet three criteria. The first criterion is ‘Where am I going?,’ and a student’s response to it will define the learning goal. To provide valid accounts of this question, the measured competence level must be related to the learning goal. Both need to be represented on the same dimension and quantified in the same currency. For example, the information that a student ‘knows all the letters of the alphabet’ would be less relevant for defining the learning goal than ‘having a good command of arithmetic in the range up to 20’; however, the information that a student can ‘bridge to ten’ certainly would. This step might be trivial for the well-defined and specific learning goals, but can present a challenge for the complex and multifaceted learning goals, such as ‘writing an argumentative essay’ or ‘being able to apply trigonometric functions to everyday problems.’ In the context of writing a good argumentative essay, for example, one may enquire about the requisite skills and knowledge. The answer would be that one needs to know about text structure, data collection, thesis development, presentation of well-supported (counter-) claims, and presentation of conclusions against the backdrop of logical, rhetorical, and statistical rules and conventions. Assessing and giving feedback about all these aspects is far from being trivial. The second question is ‘How am I going?,’ and embraces the feedback aspect of the formative assessment. This requires a semantic interpretation of the attained competence level that is plausible, legitimate, and accurate. The implications offered based on this level must be appropriate, too. Due to a lack of training in test theory, it is unlikely that all the students and teachers will arrive at a common interpretation when confronted with a single score in a competency domain. However, even if the students and teachers are formally trained in test interpretation, most decisions made in classrooms and other real-world settings usually tend to be based on holistic qualitative assessments (e.g., Moss, 2003; Stiggins, 2005; Kane, 2006). It is not difficult to imagine that information from isolated formative assessment that is not compatible with the prevailing holistic appraisal will likely be discounted or disregarded at all. This bias poses a most serious threat to the validity of the formative assessment. A similar argument can be made for the third question: ‘Where to go next?’ However, in this case, other aspects seem more relevant. The ultimate function of the formative assessment is to adjust teaching to the students’ competence level. This presupposes that we know which pedagogical action is most appropriate and practicable, given a student’s achieved competence level. Gaining this information, however, may not be very easy, and if the differences in students’ competence levels are ignored, they may lead to decisions that recommend inappropriate pedagogical actions, seriously damaging the validity of the formative assessment (see Akers et al., 2016). This brings us to the final requirement, which is particularly important for implication inference because it links pedagogical action with learning outcome. This requirement is that pedagogical action informed by data from formative assessment results in significantly better learning outcomes as compared to pedagogical action without these data. This is a very strict validity criterion, especially in settings where instruction quality is high anyway.

## Methodological Challenges and Solutions

Obtaining information from formative assessment based on computer technology in combination with complex measurement models has some demanding methodological challenges as compared to obtaining information from other sources of information such as ordinary classroom tests or observations. However, when these challenges are met, the epistemological value of such formative assessment and its utility for making truly ‘reflective classroom-assessment decisions’ (see McMillan, 2003) is much higher. In the following, we want to examine these challenges by focusing on the inferences of scoring, generalization, and extrapolation. We will contrast such assessment with the more traditional ones and point out how they can help increase the validity.

### Scoring Inference

Objective, appropriate, accurate, consistent and bias-free scoring is the basis for valid formative assessment. To fulfill these requirements, we need clear, complete, and accurate scoring rules, and we need to ensure that these rules are implemented consistently. Ideally, we also could collect empirical evidence on the quality of the scoring rules and their implementation. To evaluate students’ performance in the classroom, teachers usually develop and apply their own, often-intuitive scoring rules (e.g., McMillan, 2003). The objectivity of such scoring largely depends on the teacher. An experienced teacher, for example, is more likely to consider all the appropriate scoring options while developing the scoring rules, compared to a less experienced teacher. Time pressures or preconceptions about students’ abilities might also influence the quality of a teacher’s use of the scoring rules (e.g., Foster and Ysseldyke, 1976; McKown and Weinstein, 2008). In contrast, computer-based assessment systems offer the advantage of objective scoring through predefined scoring rules; they score the data automatically and independently of the subjects and measurement occasions. The systematic collection of data also allows the empirical validation of the predefined scoring rules via item analyses. This procedure gradually improves scoring quality by identifying wrong or flawed scoring rules (e.g., Linn, 2006). In principle, teachers could also perform such empirical validations of their own scoring rules. However, collecting relevant data and the ability to draw generalizations based on these data may not be very feasible for teachers, given their limited time and lack of expert knowledge. A computer-based assessment system allows data collected from entire populations of students to be used to validate the scoring.

### Generalization Inference

The generalization of an assessment score is especially challenging in the context of the formative assessment. Formative assessments are extremely diverse, as they are used to assess the strengths and weaknesses of each individual student repeatedly in all sorts of educational and instructional settings (e.g., Black and William, 1998; Brookhart, 2003; McMillan, 2003; McManus, 2008). From a methodological perspective, how can we ensure that these diverse assessments result in general and comparable scores with a small margin of random errors? First, a general reference or scale is required to allow us to compare the outcomes of different assessments or assessment versions. Second, item selection needs to be guided to ensure representative sampling from all eligible items. Third, item selection should focus on students’ ability levels to minimize the random error of the assessment score.

For traditional classroom assessments, teachers usually use grades as a general metric for comparing the outcomes of different assessments. However, no universal, objective rules exist for generalizing assessment scores to grades. Often, grading is influenced by the performance of the class as a whole in the sense of a norm-referenced score interpretation. Also, teachers are completely free to adjust their grading based on their subjective interpretation of the assessment content and context. For example, they can give higher grades for an average score if they think an assessment is particularly difficult, or that students had too little time to answer all the questions properly. Thus, the comparability of grades from different assessments largely depends on the class context and how teachers interpret students’ performance in terms of grades. It also depends on the teacher’s ability and experience to assemble representative items for reliable assessments to serve as sufficient information for generalizing a score or an observation (e.g., McMillan, 2003; Smith, 2003). Depending on the target competency, the range of possible assessment items is very broad and difficult to grasp, so it might be very time-consuming for teachers to prepare targeted and reliable assessments for every single student.

Computer-based assessment systems, as noted above, can support teachers in objectifying the generalizability of outcomes from the formative assessment. Computer-based assessment systems particularly allow implementing complex measurement models, such as those based on IRT (e.g., de Ayala, 2009), which can serve as warrants for generalizing the outcomes of different item sets or assessment versions (Kane, 2006). Generally speaking, IRT models imply probabilistic predictions about responses by linking person characteristics and item characteristics by some probability function. The family of Rasch models is a special case of IRT models (see Mellenbergh, 1994) and most often used in the context of educational measurement, so that we will only focus on them in the following. These models state a distinctive, monotonically increasing relation between the probability of answering an item correctly and its difficulty alongside student’s ability. One important feature of Rasch models is the underlying assumption of parameter invariance (e.g., Rupp and Zumbo, 2006). Parameter invariance holds that the assessment outcome (i.e., the ability estimate) is independent of (a) the specific items from the range of generalization chosen, (b) the order in which they are presented, and (c) the respondent. Hence, under the (falsifiable) condition that all eligible items refer to the same underlying unidimensional construct, it is possible to provide scores on a common unidimensional scale (e.g., Kolen and Brennan, 2014, p. 191), even though students work on different tailored item sets. These generalized scores are not only comparable among students but also within students across different time points. The transformation from students’ observed scores on an item level to a generalized ability score is determined by the underlying model, and is completely standardized across all assessment occasions (Wainer and Mislevy, 2000). Rasch models also serve as a tool for gathering empirical evidence to validate the model assumptions, which are crucial for generalizing the scores of various assessments, including the relation between person characteristic and item characteristic, unidimensionality, and parameter invariance.

Computer-based assessment systems, in tandem with complex measurement models, can also support teachers and students in selecting representative item samples for assessments. Ideally, such systems would include calibrated item banks. These are large pools of independent assessment items with an associated item metadata, such as item difficulty or affiliation to a content domain of the curriculum. Based on this metadata, teachers and students can identify suitable items for creating their own customized assessments, and then decide what they intend to assess and when and how to collect feedback relating to their specific questions (McMillan, 2003; Hattie and Brown, 2008). This autonomy is very important to encourage the parties to accept formative assessments (e.g., Hattie and Brown, 2008). At the same time, test blueprints and item-selection algorithms can help teachers and students select representative items and create reliable assessments. Calibrated item banks can also serve as a basis for administering computer adaptive tests (CAT; Wainer, 2000; van der Linden and Glas, 2010)—an automated form of tailored testing. With CAT, adaptive algorithms use preliminary ability estimates during test taking to select the most suitable items for each individual. These targeted items not only have the advantage of not overly demotivating students by being too easy or too difficult, but they are the most informative with regard to students’ ability. The resulting increased measurement efficiency is especially relevant if the target population is heterogeneous and/or testing time is limited. Thus, CAT contributes to the generalizability of assessment results by minimizing the random error (e.g., Lord, 1980; Wainer, 2000; van der Linden and Glas, 2010). In conclusion, we argue that calibrated item banks, based on item response theory, are an ideal tool for addressing reliability and validity. They are particularly useful because they are well adjusted to the context of formative classroom assessments (Brookhart, 2003; McMillan, 2003; Moss, 2003; Smith, 2003), and give teachers sufficient leeway for making decisions that best suit their circumstances. Also, a large item bank is a practical prerequisite that allows setting up formative assessments as a genuine process, as opposed to being a one-off event or a short-term initiative.

It is vital that data fit the proposed model and its assumptions sufficiently well. This can pose a particular challenge when students’ competency levels need to be linked across the grade levels. It is imperative then to look beyond single item fit statistics and focus instead on global fit statistics. To do so, several methods have been suggested, including those specifically developed for item response theory models (see Suárez-Falcón and Glas, 2003) as well as those borrowed from structural equation modeling (see McDonald and Mok, 1995). Models with different dimensionality assumptions should be compared against each other. Principal component analyses should also be applied to the residuals from a one-dimensional model to enable the examination of the degree to which multidimensionality is present (see Chou and Wang, 2010).^1^ In practice, it is time-consuming and costly to find adequate items that span abilities across grade levels and still meet the assumption of unidimensionality.

### Extrapolation Inference

A score that meets the requirements of scoring and generalization is meaningful only if it can be extrapolated to other competencies. From a methodological perspective, extrapolation requires three techniques. First, it requires supporting and evaluating the representative item selection. Second, it requires detecting and preventing construct-irrelevant variability. Third, it requires collecting information about assessment participation and context. Some traditional classroom assessments might fulfill these requirements while others may not. Teachers normally develop assessments and provide feedback that are closely related to their teaching (Brookhart, 2003). Thus, teaching and assessments focus on the same target competencies. However, teachers do not always have the opportunity to empirically validate whether the assessment is representative of the target competencies or whether it is unaffected by construct-irrelevant sources of variability. This might be a minor problem if the target competency is specific and well-articulated but less so for broader constructs. Regarding the extrapolation of assessment results to higher aggregated levels, teachers are usually in an ideal position to comment on the underlying student sample of an assessment group mean. For example, some students might be excluded from an assessment due to individual learning goals or simply miss the assessment because of illness. Thus, only teachers can place the aggregated values into context and interpret their true meaning. Similarly, teachers are in a favorable position to track and evaluate their students’ learning progress longitudinally, whereas it might be difficult for external parties to rely on a snapshot of available data to distinguish ‘good’ from ‘limited’ progress.

Within an item-banking system, item-selection algorithms and test blueprints can help teachers to create representative assessments by guiding the item-selection process and reverting to content specifications. Such a system can facilitate tracking previous assessments and visualizing possible gaps in content coverage in all the previous assessments. An underlying unidimensional IRT model, such as the Rasch model, can further enhance the extrapolation from the ability scores to the related competence levels, brought about by the common scales for abilities and difficulties. This relation serves as a basis for criterion-referenced score interpretation (Moser, 2009). In particular, a mastered item content or example item with a high probability can be used to map and describe a specific ability level (Beaton and Allen, 1992; Huynh, 1998). IRT models can also be used to test the construct-irrelevant sources of variability—also known as differential item functioning. This test involves correcting deviations of the probability for solving an item correctly in different groups (e.g., boys and girls), conditional on the specific ability levels in these groups (Camilli and Shepard, 1994), and providing a clear indicator of bias in an item (Lord, 1980). Construct-irrelevant variability can be minimized by targeted assessments or CAT. The administration of the easy items to low-ability students and the more difficult ones to high-ability students might prevent students from getting discouraged or bored by items that do not fit their ability levels (Asseburg and Frey, 2013). Computer-based assessment systems collect and visualize information about the participating student samples, which allow teachers and other stakeholders to use aggregated scores to draw informed conclusions about the competence levels of groups or classes. Such systems have other advantages, too. For example, they enable the longitudinal comparability of assessment results, and provide graphical illustrations of students’ learning progress; they also present empirical data about the anticipated learning progress, giving teachers, students, and external parties a broader perspective of students’ progress.

## Practical Requirements of Formative Assessment Systems

Due to its nature and scope, the formative assessment requires a huge item bank. The costs of such a bank, however, can only be reasonable if it is delivered to a large number of students. Hence, the objective of making learning visible in day-to-day school life almost inevitably turns into a large-scale project that poses practical challenges. In this section, we will introduce a developing computer-based formative assessment system to serve a population of more than 100,000 students in some German-speaking parts of Switzerland. We will highlight five practical challenges, namely item development, item calibration, item banking, assessment literacy, and ethical considerations.

### A Computer-Based Formative Assessment System

We have developed a computer-based formative assessment system^2^ to provide students and teachers with an item bank in four school subjects: German (the school’s medium of instruction), English and French (the two foreign languages taught), and mathematics. A distinctive feature of this system is its capability to cover topics and competencies from the third grade in the primary school until the third grade in the secondary school, spanning 7 years of compulsory schooling. The item bank is based on a competency-based approach to learning (see Sampson and Fytros, 2008) that emphasizes learning progress and learning outcomes during the learning process. All items used are embedded in the curriculum (see Shepard, 2006, 2008; Shavelson, 2008). Currently, the item bank contains between 4,000 and 12,000 items per school subject; up to 15,000 items per school subject have been planned for the final stage of the project.

Our assessment system has two thematically identical types of item bank: (a) the practice item bank, and (b) the testing item bank. The *practice item bank* is openly available to all the students and teachers for training and teaching purposes. Students can autonomously use this item bank to create and answer an item set from a topic domain they choose or are instructed to choose. This can virtually be done from any place that has an Internet access. Students receive detailed feedback showing which items they answered correctly, and how well they have mastered the topic in question. This item bank is also open to teachers for instruction purposes without any restrictions.

The *testing item bank*, on the other hand, can be used to evaluate students’ ability and learning progress and to identify their strengths and weaknesses in a given content domain. Teachers can select items according to the desired competency domains, single competencies, or curricular topics; they can also create tests that can be taken by students on computers at school. There are three ‘use cases’ for this item bank with three different types of feedback. First, teachers may want to use a general *competency domain*, such as reading comprehension or algebra, to assess their students’ ability or learning progress. Second, teachers can test their students on a *single competency*, such as comprehension of simple discontinuous texts or summation in the number range of a million. Finally, teachers can administer tests on *topic-specific knowledge* to assess students’ level of mastery. Such topics usually are very narrowly defined and often refer to the content of single instructional units. As opposed to the practice item bank, the testing item bank results are kept confidential in all three use cases, and students are not supposed to receive any help when trying the items. These restrictions are necessary because test results are used to automatically calibrate the item bank in terms of item-difficulty parameters.

Our formative assessment system provides performance feedback at the aggregate level of students and classes. This system can be used to promote a formative approach to instruction to support both students and teachers in setting up learning goals and monitoring their attainments (see Maier, 2015; van der Kleij et al., 2015). It has several features. First, both item banks are available throughout the school year (including break times) and hence allow for continuous monitoring of students’ ability levels and their development over time. Second, the system’s mathematical model is based on the Rasch model (e.g., Rasch, 1960), the most basic item response theory model, to determine and compare students’ ability levels on a metric scale from grade three onward, providing long-term, diagnostic learning trajectories. The Rasch model also facilitates the implementation of adaptive testing algorithms in the assessment system (see Wainer, 2000; van der Linden and Glas, 2010) as well as a fine-tuning calibration of the item difficulty parameters on a running system (see Verschoor and Berger, 2015). Finally, because all the items were developed using the formal competency-based curriculum, our formative assessment system is capable of providing criterion-referenced test scores. Thus, the feedback contains not only abstract test scores, but also tangible examples of the students’ competence levels that should help them and their teachers formulate meaningful and appropriate learning goals for each subject.

### Valid Content Specifications for Item Development

The core of an item bank for the formative assessment contains thousands, or even tens of thousands, of assessment items. Although teachers usually focus on a specific content area, substantial effort has been expended in developing items to offer students and teachers a wide range of choices. Clear content specifications are crucial for any assessment system to make valid inferences from assessment results (Webb, 2006). However, curricula or content standards, which serve as a theoretical basis for test-content specifications, often lack empirical validation (Fleischer et al., 2013). An assessment system’s empirical data contribute to the validation of the theoretical framework and the quality of the assessment items. At the same time, the theory-based content specification allows validating the decisions taken during item calibration, e.g., the selection of an IRT model or a specific linking procedure. The challenge, however, is that neither the theoretical framework nor the empirical data are completely bias-free; both sources are important for verifying each other to establish a valid scale for representing students’ genuine abilities.

We used the formal competency-based curriculum as a content framework for item development. The curriculum contains detailed descriptions of students’ competence levels, including statements about the development of each level. To put this theoretical framework into practice, we collaborated closely with content experts to develop the items for our item bank. We trained the content experts in test theory and familiarized them with our psychometric and technical guidelines (e.g., item types, number of distractors, styling). These guidelines are an important addition to the content specifications to ensure consistency within the item bank, that the items fulfill the assumptions of the underlying measurement model (e.g., measurement invariance or unidimensionality), and that they meet the system’s technical requirements (e.g., available item formats or automated scoring). More than 25,000 items are currently available in our formative assessment system. Considerable effort is needed to validate the match between the theoretical content specification of the items (i.e., their affiliation with specific competence levels in the curriculum) and the empirical, item-response-theory-based item-difficulty estimates. This validation process allows us to detect problematic items, provide feedback to our item developers, and verify our psychometric strategies.

### Item Calibration

A general scale is a prerequisite for a flexible item bank. This scale allows representing item parameters independently of a single test or predefined test versions. A vertical scale is required to measure a student’s ability longitudinally (i.e., over several school years), and provide feedback on a long-term learning progress (Tong and Kolen, 2007; Carlson, 2011; Kolen and Brennan, 2014). Unlike a horizontal scale, a vertical one combines item sets of varying average difficulty. Only a vertical scale can provide a panoramic view (7 years in our model) of a student’s ability range. A vertical scale is also a precondition for comparing ‘students’ growth in terms of criterion-referenced magnitude,’ ‘out of level testing’ by means of CAT, setting ‘proficiency cut points coherently during standard setting,’ and ‘evaluating [the alignment of] standards, curriculum and instructions, and assessment […] across grades’ (Dadey and Briggs, 2012, p. 8). As far as IRT is concerned, various calibration and linking strategies have been introduced to establish a vertical scale (see Kolen and Brennan, 2014, for a general overview). The challenge here is to identify a calibration design and strategy that corresponds to the size of the available calibration sample, and is compatible with the properties of the measured construct and definitions of growth (i.e., domain vs. grade-to-grade definition of growth) (Kolen and Brennan, 2014).

The calibration of potentially tens of thousands of items in a computer-based item bank is a highly resource-intensive process. To establish vertical scales, we developed a common-item, non-equivalent group design (Kolen and Brennan, 2014). This strategy helped us to calibrate a few hundred anchor items, representative of target competencies and target grades. The calibration design, in more specific terms, consists of a combination of grade-specific and linking items. Grade-specific items are administered to one specific grade cohort only, whereas linking items are shared between two adjacent grade cohorts (Berger et al., 2015). This way, we managed to lay the foundation for establishing a link over different target grades and relating the items to one underlying vertical measurement scale. We then exported the response data to calibrate the anchor items; we did so using the Rasch model (Rasch, 1960) by means of marginal maximum likelihood (MML) estimation procedures. The calibrated items will subsequently serve as anchors for locating additional, uncalibrated items on the scale by means of online calibration (Verschoor and Berger, 2015). For new items with no or very few observations, an Elo update scheme (Elo, 1978) was used to determine the preliminary difficulty estimates of the items. The online-calibration algorithm, in its next move, will automatically switch to a joint maximum likelihood (JML) estimation process (Birnbaum, 1968). Thanks to online calibration, we can start the system after a brief offline calibration phase, which involves extending the item pool and improving the parameter estimates systematically, while students and teachers engage with the system.

### Item Bank Development and Maintenance

The development and maintenance of the item bank, i.e., the ‘organized collection of items’ (Vale, 2006, p. 268), also pose some challenges. Computerized item banking is crucial for inventorying thousands of items, locating relevant items, tracking item usage, and developing an item’s state or life cycle. In an item bank, the item content is stored in a respective metadata on the item properties, e.g., a unique item identifier, content classification, scoring key, or the name of the item’s author. Additional item properties are based on the empirical data, such as IRT parameters or item exposure. Items can be classified in the item bank by their development state (e.g., new, calibrated, retired) and their relation (i.e., social order) to other items in the item bank (e.g., friend items, which must always appear together or enemy items, which must not be used in the same test; see Vale, 2006). All this information supports item-bank users in item selection and scoring; it is especially relevant when the system itself is responsible for automated item selection and scoring in CAT. However, CAT does not solely rely on an organized collection of items with relevant item properties, such as IRT parameters and content classifications. CAT can provide reliable and efficient ability estimates only if the item bank consists of a sufficient number of items relating to the target competencies and if item overexposure is prevented (Veldkamp and van der Linden, 2010; Thompson and Weiss, 2011). An item-banking system can help psychometricians to use simulation studies to evaluate the fit of the available items ahead of item administration.

In our formative assessment system, we use also the item bank for helping teachers and students to identify the relevant items for constructing their own formative assessments. For this purpose, teachers and students have access to selected item properties within the item bank. In particular, they can filter the contents the item bank in two ways. They can use content categories, namely the curriculum competence levels and related topics, or filter items in relation to the vertical scale, which represents the difficulty of the items on the same scale based on the reported scores. Thus, the outcomes of previous assessments can guide targeted item selection. Additional item properties are automatically used by the system to support teachers and students in constructing sensible assessments. For example, the system informs users about friend items (Vale, 2006), such as listening-comprehension items that are related to the same audio text. The identification of friend items helps teachers and students to create more authentic assessments; this way the students can answer multiple items related to the same support material, rather than switching the context after each item. This is especially relevant in competency domains such as reading and listening comprehension, in which processing the support material during test taking (i.e., reading a text passage or listening to an audio file) can be rather time-consuming.

### Technological and Organizational Challenges

Setting up a large-scale computer-based assessment system can inevitably pose several technological and organizational challenges. There are challenges that are purely technological or specific to the design of the human–machine interface. The technology must be capable of perfectly supporting a wide variety of systems, devices, and browsers at school, at home and on the road. Considering the fact that there lacks a central instance for keeping operating systems up to date, in practice, there are a large number of versions and update stages that require supporting. For pragmatic reasons, this limits the prospects of deploying new versions of the assessment software that would need to be extensively tested on all the various systems. As a compromise between user friendliness and practicality, our assessment system is only fully compatible with the latest two versions of the most popular internet browsers (i.e., Chrome, Firefox, Internet Explorer/Edge, and Safari). The infrastructure must also be capable of supporting several thousands of concurrent users during morning access peaks in the school. This is especially challenging for computer adaptive testing, not least because a continuous real-time communication with the item bank is required to select the appropriate items based on the students’ previous responses. To manage the load during peak periods, we implemented multiple instances of the assessment delivery module of our assessment system, which allow us to distribute the load. From a design point of view, the development of an intuitive user interface is crucial, mainly because small deviations from the optimum will immediately result in a surge of customer support requests. Design also needs to take into account the broad age range of users and their scope of digital expertise.

Practical challenges also arise in relation to populating and maintaining the item bank, the large scale of which augments the demands for accuracy and the impact of errors. With thousands of items in each domain, we needed to set up comprehensive, standardized guidelines for designing items across different subjects, content domains, and different school grades or age groups. Quality assurance in a huge item pool is also challenging and labor-intensive: typing errors and errors in the scoring key need to be detected and eliminated, psychometric properties of the items should be constantly monitored, conspicuous items ought to be flagged and double-checked, and content specification needs to be consistently checked to ensure that items are assigned to the most suitable content category within a growing item pool. The maintenance of the item bank also requires a constant investment of time and effort. The item development outside the system needs to be synchronized with the active item pool, and updates of the items need to be carefully integrated into the system. To do so, it is necessary to keep track of the item versions and to decide whether or not updates need to be applied to the item parameters. Subsequently, eliminated items need to be replaced with new ones and matched to the content domains based on the difficulty level.

The quality assurance requires that all data be exported on a regular basis for an offline quality control. This quality control comprises the analysis of item discrimination parameters, a distractor analysis, an investigation of the item fit, and an analysis of differential item functioning between different school grades and types. From a practical point of view, we need to ensure that the data export does not interfere with system performance; that is why it usually takes place outside the usual working hours. We also need to ensure that the export meets all the standards of privacy and data protection. In the future, most of the quality assurance will be implemented automatically within the system to limit the need of data export. This, however, requires even more testing and supervision until the online quality assurance runs flawlessly. We decided to invest this testing and supervision effort and hope that it will pay off in the long run.

A final challenge that deserves a mention, although in passing, concerns designing reporting materials that support a valid interpretation of the results by students of all grades and at all stages of cognitive development. Although there are guidelines and even studies that have investigated design principles for assessment reports, few recommendations exist for age diverse populations. We have needed to adapt our materials several times and are now planning to run randomized controlled trials to investigate which type of report is best understood by whom.

### Challenges Concerning Stakeholders’ Assessment Literacy

Consequential validity (Messick, 1989, 1995; Kane, 2006, 2013), as the core aspect of the implication inference, strongly requires that all feedback be appropriately understood and interpreted within an inevitable margin of error. In the extant literature, this issue is referred to as ‘assessment literacy,’ and is defined as the ‘understandings of the fundamental assessment concepts and procedures deemed likely to influence educational decisions’ (Popham, 2011, p. 267). Popham emphasizes three important aspects in this definition. First, ‘understanding […] concepts and procedures’ does not necessarily imply that assessment users are able to develop and run reliable and valid assessments by themselves; equally, they may not know how to calculate ability estimates, standard errors, or reliability coefficients. However, users are expected to recognize the concepts and procedures, and know what they mean to arrive at valid interpretations of them. The focus of the second aspect is on ‘fundamental’ concepts and procedures, which encompass knowledge that is just about enough and necessary in the respective applied context. Hence, users are not expected to understand the different ways of calculating the different reliability coefficients. However, they should, for instance, understand why a reliability of ρ = 0.50 is by far not enough for the interpretation of individual test scores. Popham (2009) has proposed 13 ‘must-understand topics’ for teachers and administrators. One example is the understanding that the function of educational assessment is ‘the collection of evidence from which inferences can be made about students’ knowledge, skills, and affect’ (p. 8). Third, the understanding inherent in the concept of assessment literacy is limited to concepts and procedures that are ‘deemed likely to influence educational decisions.’ Assessment literacy, as defined above, does not imply that users understand all aspects of assessment but only those that are relevant to everyday decisions. Each of these three points is highly compatible with the concept of consequential validity advanced by Messick (1989, 1995) and Kane (2006, 2013).

There are three more aspects of assessment literacy that have received relatively limited attention. The first aspect is in line with the modern notion of competencies (see Klieme et al., 2008). It refers to the non-cognitive facets of assessment literacy, such as attitudes toward measurement, beliefs about one’s own efficacy to make useful decisions based on assessment results, or motivational factors associated with their use. These non-cognitive facets interact with the cognitive ones. A basic understanding of the fundamental assessment concepts and procedures can cultivate high self-efficacy beliefs and positive attitudes toward educational measurement. In turn, these positive beliefs and attitudes are expected to facilitate the understanding itself. Indeed, there is some evidence that holistic assessment literacy programs that look to assessment literacy as an integral part of professional development are more effective than programs that focus on technical and methodological aspects only (e.g., Koh, 2011). Such programs are probably key to using assessments appropriately. If teachers are extensively supported in conducting, analysing, and interpreting their assessments and learn to relate the assessments to the taught content, chances are good that they will accept formative assessment as a valuable tool in their work, start using it on a regular basis, and develop a sense of self-efficacy when using it.

Second, assessment literacy requires a positive assessment culture in which the process of the formative assessment follows certain requirements, such as the application of intra-individual standards of reference. Black and William (1998) also stress the importance of interaction and dialog in instruction to promote opportunities for students to express their understanding and for teachers to evaluate it. The Assessment Reform Group (1999, p. 7) argues that assessment is more likely to promote learning if it (a) is embedded in a view of teaching and learning of which it is an essential part, (b) involves sharing learning points with students, (c) aims to help students learn and recognize the standards they aim to achieve, (d) involves students in self-assessment, (e) provides feedback that informs students of subsequent action points, (f) is underpinned by confidence that every student can succeed, and (g) if it involves both teachers and students reviewing and reflecting on assessment data. Collectively, these points emphasize a positive and collaborative assessment culture that is a fundamental part of instruction (points a, f, and g), in which students and teachers are not only actively involved but also empowered to draw their own conclusions about their learning processes (points b, c, d, and e).

The third aspect concerns stakeholders’ involvement, mainly students and teachers, but also administrators, test developers, and researchers with varying educational backgrounds, interests, and motivations. Teachers need to be assessment-literate to understand the scientific approach to educational measurement and the benefits of the use of formative assessment. Their assessment literacy should at least comprise the key elements of the assessment process, sometimes portrayed as the assessment triangle, comprising ‘a model of student cognition and learning in the domain, a set of beliefs about the kinds of observations that will provide evidence of students’ competence levels, and an interpretation process for making sense of the evidence’ (Pellegrino et al., 2001, p. 44). Although there is evidence that teachers’ assessment literacy is linked with notable benefits in students’ learning (e.g., Wilson et al., 2001), studies suggest that currently teachers’ competence levels in this regard are mediocre at best (Mertler, 2004; DeLuca and Klinger, 2010; Popham, 2011). Similar findings have been reported about teachers’ self-described levels of assessment self-efficacy and literacy (e.g., Volante and Fazio, 2007). This is hardly surprising, considering the limited role of assessment literacy in teacher-education programs (e.g., DeLuca and Bellara, 2013). In an extensive review of measurement textbooks, Shepard (2006) found limited guidance ‘about how teachers were to make sense of assessment data so as to redesign instruction’ (p. 625). Teachers’ lack of assessment literacy is likely to pose a serious and hardly controllable threat to validity in formative assessments, despite the existence of several initiatives and interventions to promote teachers’ assessment literacy (e.g., Wang et al., 2008; Xu and Brown, 2016).

Students need to be assessment-literate as well to incorporate feedback in their learning processes adequately and get valid answers to Hattie’s fundamental questions: where to go, how to get there, and where to go next (Hattie and Timperley, 2007). Equally important are their metacognitive strategies and self-regulation skills, which can be promoted by a competent utilization of formative assessment (Nicol, 2009; Sadler, 2009). Despite the growing interest in and application of testing and formative assessment in schools, there is a paucity of research dealing with this aspect of assessment literacy. However, one can assume that young and/or underachieving students might become overstrained by the demands of complex assessments. Francis (2008), for example, argues that even first-year university students tend to overrate their understanding of the assessment process. Programs that aim to promote assessment literacy in students exist (e.g., Smith et al., 2011), but they are usually targeted at adolescents or young-adult students, and to the best of our knowledge, no program exists for younger children.

### Considerations on Ethics and Privacy

The potential benefits of this technology need to be evaluated against the potential ethical concerns that may arise from its usage. The first concern regarding computer-based formative assessments relates to *trust* (e.g., Lee and Nass, 2010). This is particularly crucial when students and teachers make consequential and potentially long-term decisions based on (necessarily) imperfect results. We partially have addressed this issue when discussing the necessity of assessment literacy for understanding and interpreting assessments, but the concern is broader. Computer algorithms might fail and produce flawed outcomes for longer periods of time before being detected. Students and teachers might overestimate the reliability and validity of the results that are neatly presented and appear to be backed scientifically. This may cause disappointments, especially if these expectations are unduly high.

The second ethical concern is the risk of discrimination (see Datta et al., 2015). It is widely recognized that learning algorithms are prone to biases (Caliskan et al., 2017) so that extreme care needs to be put into the selection of algorithms and the interpretation of their results to ensure that these biases are not projected (and possibly exaggerated) by the feedback provided. The nature of this problem is fundamentally different from the *correctness* of results noted above. Here, while results may be considered correct, they may slightly differ for different subjects, hence the discrimination. On the same note, one might also be concerned about the fairness of enhancement (e.g., Savulescu, 2006). If students with greater aptitudes, higher motivation and/or easier physical access to the system benefit more from it than their peers of the reverse profile, formative assessments could widen the existing social discrepancies in education rather than narrowing them. Whether this concern is reasonable or not needs to be scrutinized in carefully designed empirical studies that track students’ learning progress over time, control for any endogeneity bias, and consider the didactic method of teaching. Some didactic setups indeed might widen existing gaps, while others might do the opposite.

The collection of previously unexamined data in educational environments may lead to unintentional leaks about students and/or teachers. These accidental discoveries may range from trivial matters, such as secret friendship between two students (e.g., when log-in times and selection of items are correlated for two students), to more serious affairs, such as bullying or family disruption (e.g., when sharp declines in performance are detected and cannot otherwise be explained). While well documented in the medical research, the manner of dealing with such incidents is yet to be explored in the domain of formative assessments. Finally, the creation of large databases about students’ knowledge and beliefs at such a young age raises concerns regarding the potential dual use of these data. While the term ‘dual use’ has been traditionally used for technology—designed for civilian purposes but with potential military applications—we believe that the recent revelations such as the Cambridge Analytica case illustrates that the capacity for data misuse exceeds the boundaries of this definition. In summary, it is extremely important to carefully consider the manner in which data are collected and disseminated.

In addition to ethical considerations, privacy issues arising from data collection are a serious concern in all kinds of computer-based assessment systems, and even more serious as systems grow both in scale (i.e., the number of students) and scope (i.e., the amount of data, also known as ‘big data’). The existing guidelines, however, are surprisingly silent on data protection and privacy. The International Test Commission (2006), for instance, defers to ‘local data protection and privacy legislation’ (p. 166), whereby most systems incorporate instances of privacy management (e.g., Plichart et al., 2004). We believe that there are two major issues that must be taken into account here. First, when building computer-based assessment systems, a careful consideration of the regulations dealing with the protection of personal data (e.g., GDPR in Europe or COPPA in the United States) is crucial. These legislations address issues that have an effect on how technology has to be designed and deployed. They require, for example, clear statements respecting the nature of the data collected, the purpose for which they have been collected, strict control on individuals who can access the data, the acquisition of consent (parental consent in case of minors), and transparency of data treatment within the system. The intricate educational ecosystem alongside the complexity of algorithms used make some of these tasks extremely difficult.

The design of computer-based assessment systems should always take privacy seriously. Formative assessments, as noted earlier, make learning visible not only to students or teachers but potentially to all parties involved. Also, special caution needs to be exercised when assessment data are being matched with other sources of data (e.g., socioeconomic status or language spoken at home), especially when individual students become identifiable. Indeed, large-scale, computer-based assessment systems must deal with the inherent dilemma between privacy and the right to self-determination over one’s own data. However, there is a scientific and administrative desire for rich and abundant data for research and administrative purposes. Thus, care has to be taken that the data collated are strictly necessary in use and exposure. This in some cases may be achieved using advanced privacy-enhancing technologies, such as the processing of encrypted data or anonymization of communication. How to integrate these protection technologies in the workflow of educational tools is a promising subject for future research.

## Conclusion and Outlook

In this paper, we discussed the epistemological, methodological, and practical aspects of computer-based tools for formative student assessment, which aims to support learning and data-based decision making. In view of the effects of formative assessment and the benefits of data-based decision making, we are convinced that such tools can offer many advantages, compared with more traditional ways of providing feedback and making educational decisions. From an epistemological perspective, the most compelling advantage lies in the anticipated improvement of validity in computer-based tools, compared with feedback procedures based on teacher intuition and other unsystematic approaches. We have argued that these improvements can extend to all levels of the interpretive argument, ranging from scoring to generalization, extrapolation, and interpretation of results. Obviously, it is difficult to quantify these improvements in advance; however, given the number of aspects involved, one can assume that the scope of improvement will be substantial.

A second advantage of computer-based tools for formative assessment and data-based decision making is their considerable potential for enhancement in terms of availability, versatility, and flexibility at a small cost (in terms of organization and time) for the teachers and students involved. They provide options on the length of assessments, the time of administration, and competencies or topics that are currently relevant. Teachers, for example, can offer them to all their students or only to those whom they consider to be the most in need. Students can choose to run assessments on a regular basis or when they feel that one is necessary. These versatility and flexibility features are a direct function of the size of the item bank; however, once the curriculum has been covered in sufficient breadth and depth, the combinatorial prospects of creating tests can grow considerably.

Computer-based formative assessments have further advantage. They may be used to alleviate social disparities in learning and allow weak students to benefit from an idiosyncratic standard of reference. They can positively influence instruction by improving teachers’ curriculum orientation and systematic planning, and contribute to promoting a positive testing culture in schools, in which assessments are not regarded as an external threat, but rather as a beneficial tool.

A flawless, state-of-the-art computer-based tool for the formative assessment needs to keep pace with the current massive technological advancements. Three developments are likely to influence what such systems will look like in the future. The first is the implementation of innovative item formats with interactive elements that allow assessing students’ productive competencies (see Goldin et al., 2017). Such items could contain simulations of conversations with interactive chat bots, writing assignments that are automatically scored with respective algorithms, or geometrical construction tasks with interactive elements. All these would make full use of the computer-based platform and allow assessing both outcomes and the problem-solving processes.

The second potential enhancement resides at the methodological level. By using information on both learning processes and outcomes and reverting to this ‘big data,’ constantly produced by the system, one could start using such systems as tools for cognitive diagnostics and learning analytics. Cognitive diagnostics instruments enable an in-depth assessment of students’ competence levels and automatic presentation of items and tests following suggestions offered based on the collated empirical evidence; these data about the competencies are needed to answer the items and understand how these competencies relate to each other for each individual student. These relations could use cognitive models (e.g., Frischkorn and Schubert, 2018) as a starting point and be further refined by means of automated experiments so that the algorithms could learn by themselves what works best for which students and when. All this is closely related to the concepts and methods put forward in the emerging field of learning analytics (see Siemens, 2013). Here, there is also the idea to discover hidden relations in data but the focus is more on informing and empowering teachers and students about the learning process. A case in point are systems such as the ‘Course Signals’ at Purdue University (presented in Clow, 2013) that are used to predict success and failure in specific courses based on demographic characteristics, previous academic history, interaction with the system itself and performance on the course to date. This can be done very early during the course and as a consequence, instructors can trigger several interventions meant to prevent failure. Formative feedback systems such as the one introduced above are perfectly suitable as a rich data source for this kind of applications.

Third, given the growing importance of lifelong learning and the popularity of informal learning, it is unlikely that the future of computer-based formative assessments will remain restricted to schools and other educational institutions. This trend is likely to promote personalized learning environments, potentially available to everybody and for a broad range of topics. Combined with innovative and appealing item formats and supported by powerful diagnostic algorithms, we may eventually arrive at truly intelligent tutoring systems that are well-integrated into our daily lives.

## Author Contributions

UM developed the concept and chaired the practical implementation of the formative assessment system, MINDSTEPS, used here as a sample case. UM and SB were equally involved in developing its methodological foundations. MT drafted the article based on contributions by all authors, particularly UM who wrote on the theoretical background of formative assessments and SB who focused on the methodological and practical issues. All authors have revised the draft and approved the final version to be submitted.

## Conflict of Interest Statement

The formative assessment system used as a case in point in this study was commissioned by the Bildungsraum Nordwestschweiz, which funded its development and operation. The authors have disclosed to the article’s editor the details of the financial relation between the initiative and the sponsoring institution.

## Footnotes

1 We are grateful to the reviewer for drawing our attention to this issue.

2 https://www.mindsteps.ch/
